# Supplementary material for: Xyloglucan–Cellulose Nanocrystals Mixtures: A Case Study of Nanocolloidal Hydrogels and Levers for Tuning Functional Properties
Source: Gels. 2024 May 15;10(5):334. doi: 10.3390/gels10050334 (PMC11121706; doi:10.3390/gels10050334)
Supplement: Supplementary file 1 [file gels-10-00334-s001.zip › gels-3005220-supplementary.pdf]

Xyloglucan-cellulose nanocrystals mixtures: a case study of nanocolloidal hydrogels and levers for tuning functional properties

Géraldine Rangel[1], Céline Moreau[1], Ana Villares[1], Christophe Chassenieux[2], Bernard Cathala[1]*

Supplementary information

1 UR1268 BIA, INRAE, 44300, Nantes, France

2 IMMM UMR CNRS6283, Le Mans Université, Avenue Olivier Messiaen, 72085 le Mans France

* : corresponding author

email : Bernard.cathala@inrae.fr

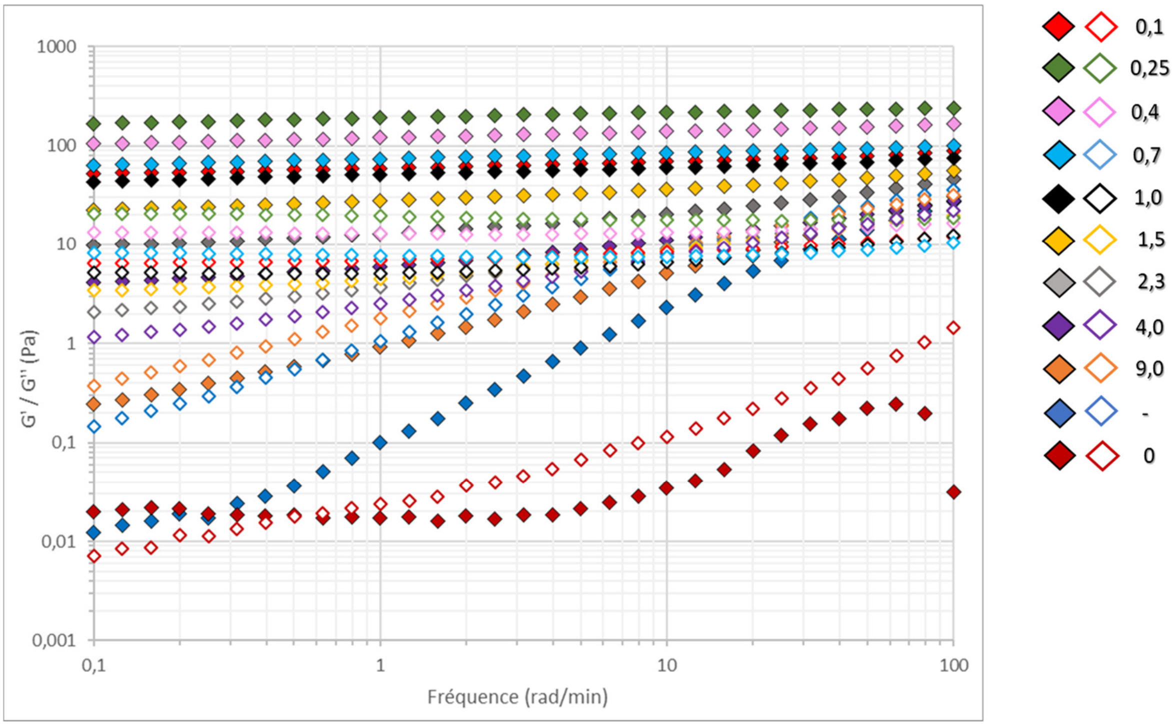

Figure S1. Frequency sweeps for the different mixtures at 20 g/L covering all range of XG/CNC ratio explored. G′ (full diamonds), G′′ (empty diamonds).
